# Economic support to improve tuberculosis treatment outcomes in South Africa: a qualitative process evaluation of a cluster randomized controlled trial

**DOI:** 10.1186/1745-6215-15-236

**Published:** 2014-06-19

**Authors:** Elizabeth Lutge, Simon Lewin, Jimmy Volmink

**Affiliations:** 1 Faculty of Health Sciences, Stellenbosch University, Francie van Zijl Drive, Tygerberg 7505, South Africa; 2 Health Systems Research Unit, South African Medical Research Council and Norwegian Knowledge Centre for the Health Services, Francie van Zyl Drive, Parrow 7505, South Africa; 3 Cochrane Centre, South African Medical Research Council, Francie van Zyl Drive, Parrow 7505, South Africa

**Keywords:** Economic, Support, Incentives, Enablers, Tuberculosis, Qualitative process evaluation, RCT

## Abstract

**Background:**

Poverty undermines the adherence of patients to tuberculosis treatment. A pragmatic cluster randomized controlled trial was conducted to investigate the extent to which economic support in the form of a voucher would improve patients’ adherence to treatment, and their treatment outcomes. Although the trial showed a modest improvement in the treatment success rates of the intervention group, this was not statistically significant, due in part to the low fidelity to the trial intervention. A qualitative process evaluation, conducted in the final few months of the trial, explained some of the factors that contributed to this low fidelity.

**Methods:**

In-depth interviews were conducted with patients who received vouchers, nurses in intervention clinics, personnel in shops who administered the vouchers, and managers of the TB Control Programme. These interviews were analyzed thematically.

**Results:**

The low fidelity to the trial intervention can be explained by two main factors. The first was nurses’ tendency to ‘ration’ the vouchers, only giving them to the most needy of eligible patients and leaving out those eligible patients whom they felt were financially more comfortable. The second was logistical issues related to the administration of the voucher as vouchers were not always available for patients on their appointed clinic dates, necessitating further visits to the clinics which they were not always able to make.

**Conclusions:**

This process evaluation identifies some of the most important factors that contributed to the results of this pragmatic trial. It highlights the value of process evaluations as tools to explain the results of randomized trials and emphasizes the importance of implementers as ‘street level bureaucrats’ who may profoundly affect the way an intervention is administered.

**Trial registration:**

Current Controlled Trials ISRCTN50689131, registered 21 April 2009.

The trial protocol is available at the following web address: http://www.hst.org.za/publications/study-protocol-economic-incentives-improving-clinical-outcomes-patients-tb-south-africa.

## Background

There is general agreement that tuberculosis (TB) remains strongly associated with conditions of poverty [1]. Poverty increases the risk of infection and disease and, through its effects on nutrition as well as its impact on adherence to treatment, undermines the outcomes of patients on TB treatment.

TB is the most common cause of death in South Africa [2] and although this is due in part to widespread co-infection with HIV, poverty still plays an important role in the continued significance of the disease [3,4]. Poverty may cause delays in accessing initial treatment for TB, thus delaying the detection and treatment of TB and its complications. Even after the initiation of treatment, the barriers that poverty imposes on access to treatment may also reduce the likelihood of treatment success [5]. In South Africa, deprivation is an important determinant of the use of primary health care services (the level at which most TB care is delivered). The primary healthcare utilization rate is lowest (between 2.0 and 2.1 visits per person per year) in the most deprived districts of the country and highest (3 visits per person per year) in the least deprived (the national target for the country at the time of the trial was 3.5 visits per person per year) [6]. Studies among South African patients on TB treatment have found that financial constraints are major obstacles to the completion of TB treatment [7,8] and TB preventive therapy [9]. Although economic interventions may assist patients to adhere to their TB treatment schedules, very few studies have been conducted to evaluate their effects [10]. Most of the trials that have investigated this took place in the United States [11], and may not be generalizable to poor- and middle-income countries where the burden of TB is highest.

This paper reports on the qualitative process evaluation of a cluster randomized controlled trial, in which the effect of economic support on the outcomes of patients with active TB in South Africa was investigated [12]. The trial itself is described below.

### Description of the trial and its results

This pragmatic, cluster-randomized controlled trial was conducted in two districts of KwaZulu-Natal, one of the poorest provinces in South Africa which has the highest burden of TB and HIV in the country. In this trial, patients with drug-sensitive pulmonary TB were offered a monthly voucher valued at ZAR120 (approximately US$15) until completion of treatment or a maximum of eight months. Vouchers were offered to patients and administered by the nurses in charge of the TB program at participating clinics. We postulated that the vouchers, if redeemed for food, would increase patients’ food security and free up money for use elsewhere, such as for transport to attend the clinic. Vouchers were redeemable at selected general stores situated close to the clinics. Patients in control clinics received usual TB care. To prevent ‘leakage’ of the vouchers to those not eligible to receive them, patients were required to present their clinic cards at the shops with their vouchers which corresponded to the clinic number on their vouchers. In addition, nurses signed each voucher, and stuck onto each voucher a unique sticker, without which the voucher could not be redeemed. The trial started on 1 July 2009 and ended on 30 September 2010, when the last recruited patients had completed their full course of treatment. All eligible patients, that is, 4091 patients were included in the trial: 1984 in the control arm (10 clinics) and 2107 in the intervention arm (10 clinics). Intention-to-treat analysis showed a small but non-significant improvement in treatment success rates in intervention clinics (intervention 76.2%; control 70.7%; risk difference 5.6% (−1.2; 12.3%), *P* = 0.107). There was a strong dose-response effect; treatment success increased with the number of vouchers received (*P* <0.001). However, fidelity to the intervention was low. Of all the patients who were eligible to receive a voucher, 813 (36.2%) did not receive a voucher at all, and 671 (32.3%) received a voucher for between one and three months. The remainder received a voucher for four to eight months of treatment.

### The value of process evaluations of randomized controlled trials

Although randomized controlled trials provide high quality evidence for the efficacy or effectiveness of an intervention, they often do not provide contextual information that might help explain the findings of the trial. Such information is particularly important in pragmatic trials where the real-world settings in which interventions are delivered may have a profound impact on the conduct and the findings of the trial. Process evaluations conducted alongside such trials can provide valuable insights on why the trial yielded such results, which is important for the replication or large-scale implementation of trial interventions [13].

Process evaluations attempt to view the trial from the perspective of the implementers and the recipients and use both qualitative and quantitative methods to analyze the contributions of a variety of factors to the effectiveness, or otherwise, of an experimental intervention [14]. Importantly, where an intervention is not effective, process evaluations can help to ascertain whether it is the intervention itself that is inherently flawed or whether the way in which it was delivered undermined its effectiveness [15].

This process evaluation focuses on how participants in the trial viewed the economic support provided to patients with TB, and how these views impacted on the conduct of the trial. It also explores participants’ opinions on the principle of economic assistance for the achievement of health outcomes, which has important implications for the implementation of, and further research into, such interventions.

In addition to helping to explain the trial results, it was important that a process evaluation be conducted alongside this trial because the provision of social welfare in South Africa (and elsewhere), including economic support to people with specific illnesses, remains a contested issue [16,17]. Specific concerns that have been expressed are that: social grants will have a perverse incentive effect (people will behave in a way that will ensure continuation of the grant; for example, patients with TB will deliberately not complete their treatment in order to continue receiving a grant for TB); people will become dependent on the grants and will not try to find work because their needs are met by the grant; and finally, people will spend the grant monies on frivolous or unhealthy items. It was important that this trial investigate and report on these issues.

## Methods

This was a qualitative process evaluation, conducted in the final few months of the trial. This process evaluation was based on in-depth interviews describing the views and perceptions of the key stakeholders in the trial.

### Trial setting

This trial, and this process evaluation, was conducted in one rural district (Uthungulu) and one urban district (eThekwini) of KwaZulu-Natal (South Africa) which have the fifth and sixth highest TB incidence of the 52 districts in the country, respectively [18]. In a deprivation ranking of all South African districts, eThekwini lies within socioeconomic quintile 4, and Uthungulu in socioeconomic quintile 2 (where quintile 5 is least deprived, and quintile 1 is most deprived) [6]. Within eThekwini, however, are large areas of deep poverty, such as informal settlements and former townships. It is in these areas of the city that the study was conducted.

### Population and sampling

**Figure 1:**
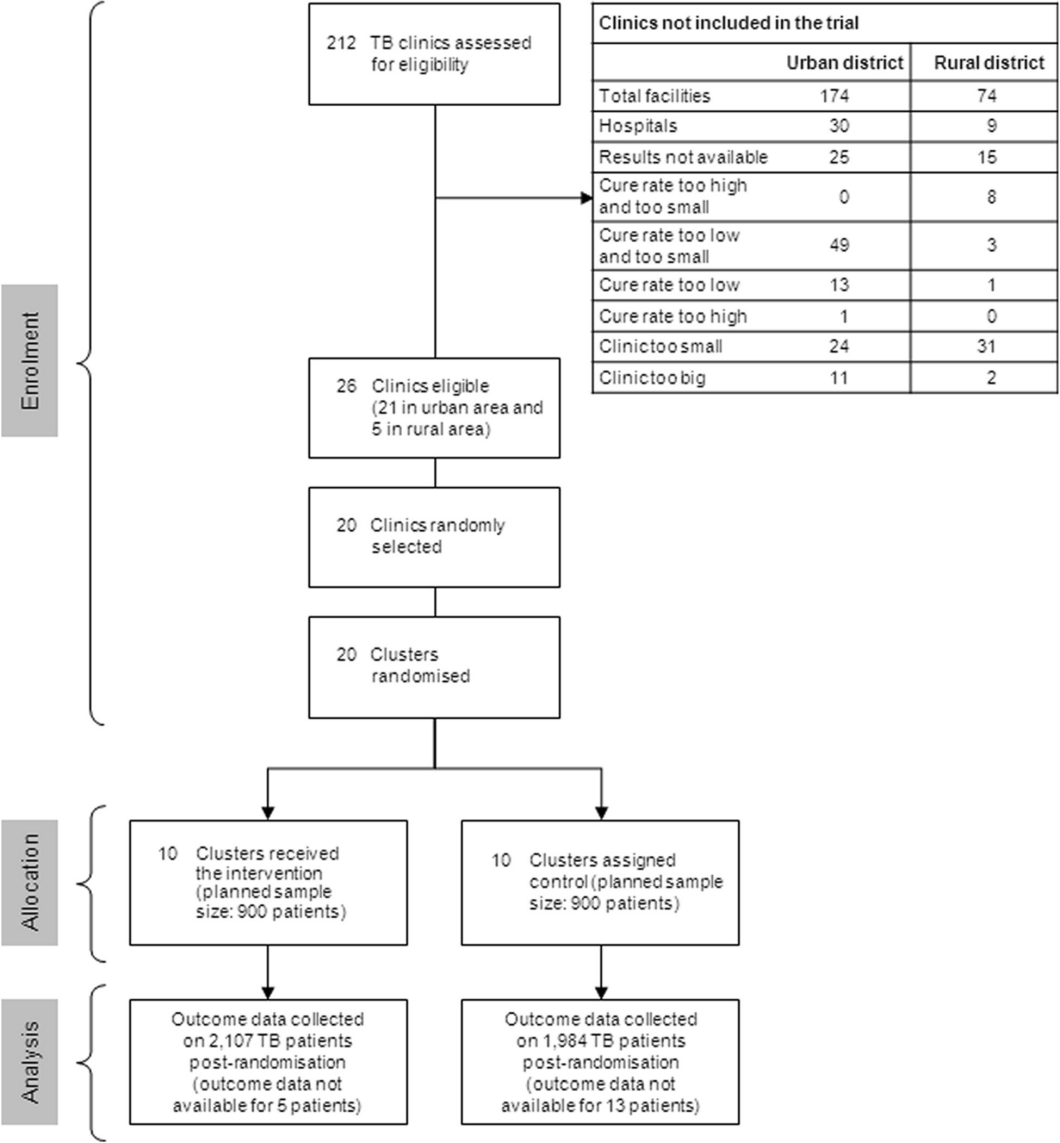
**Consort flow diagram of trial.** TB, tuberculosis.

Clinics with cure rates of between 40 and 70% for the year preceding the trial were eligible for inclusion. All patients diagnosed with pulmonary drug-sensitive TB, and attending intervention clinics within the period 1 July 2009 to 31 March 2010 were recruited into the trial and started receiving the voucher immediately, irrespective of their stage of treatment. However, only patients who started TB treatment within this recruitment period were eligible for analysis. The Consort flow diagram depicting the flow of patients in the trial is shown in Figure 1. The Consort checklist is available in Additional file 1.

Interviews were conducted with patients who had received vouchers, nurses who had administered vouchers at intervention clinics, shop owners, managers, and cashiers at the shops where vouchers were redeemed, and managers of the TB Control Programme at district, provincial, and national levels, who had been involved in or were well informed about the trial. In each clinic, the nurse in charge of providing TB care was selected to participate in this process evaluation. Similarly, in each district, the managers of the TB Control Programme were asked to participate, as was the national manager of the TB Control Programme. The manager of each shop was approached to participate but in some cases, she or he delegated a staff member to do so. All eligible patients attending the clinics within the period of data collection for this process evaluation were approached to participate.

All nurses interviewed were professional nurses, with a median nursing experience of 15 years. Patients had a median of 8 years schooling. Sixty nine percent of patients were unemployed, 14% worked in the informal sector, 3% worked in the formal sector, and 14% were too young to work. Study participants are further described in Table 1.

**Table 1:** Participant demographic information

**Group (number interviewed)**	**N (%) urban**	**N (%) female**	**Median age**	**Median level of education**
Patients (29)	18 (62)	22 (76)	35	Grade 10
Nurses (7)	5 (71)	5 (71)	40	All professional nurses
Managers in the TB Control Programme (5)		3 (60)	49	Data not collected
Shop personnel (7)	5 (71)	5 (71)	30	Data not collected

Interviews were conducted with patients and nurses at intervention clinics. Clinics were purposefully selected to represent the range of settings in the trial, so that large, small, urban, and rural clinics were included. Nurses in charge of the TB program at seven out of ten participating clinics were interviewed. At the same clinics, patients were approached to be interviewed if they were receiving the voucher and if they were attending one of the seven selected clinics on the day on which interviewers were present.

Seven out of eight shops were included in this process evaluation. This ensured that the range of stores participating in the trial (which included large chain stores as well as small owner-managed shops, from both urban and rural areas) were represented. Personnel in management positions who had been involved in the administration of the vouchers were invited to participate. Four administration managers, one general manager, one owner, and one cashier were interviewed.

Three senior TB managers from the two districts and one from the province in which the trial was conducted were interviewed, as well as one senior TB manager from the national level. These managers were selected because of their role in the running of the TB Control Programme, their awareness of the study from its inception, and because they were involved in policy formulation for TB management. No participants refused to be interviewed.

### Data collection

Interviews were semi-structured and the focus of the interviews was the administration of the voucher, the effects of the voucher on patients’ adherence to treatment, and the principle of providing economic assistance for the improvement of health outcomes.

The data collection process was iterative, in that follow-up interviews were conducted with selected participants to clarify issues raised in the initial interviews, or to discuss new issues that arose during implementation of the voucher system. Interviews were conducted until data saturation was reached.

All interviews were conducted face-to-face, audio-recorded with the consent of the interviewee, transcribed verbatim, and translated into English where necessary. Interviews with patients were conducted in isiZulu, whilst interviews with all other participants were conducted in English. Research assistants, who were trained by a qualified qualitative researcher, introduced themselves to all participants, emphasizing that they were from an independent research organization and not affiliated with the health services. Patients were assured that non-participation in the interviews would not affect their participation in the trial nor their receipt of services from the clinic. Interviews with patients lasted about 75 minutes, whilst those with nurses and TB managers lasted about an hour, and those with shop personnel lasted about 30 minutes. Interviews with patients took place in the clinic grounds away from the clinic building, or in an unused room within the clinic, to preserve patient privacy. Interviews with nurses took place in unused consulting rooms or offices at the clinics, whilst those with shop owners, managers, and cashiers took place in the administration offices of the shops. Interviews with TB program managers took place in their offices or at a designated public meeting place.

### Data analysis

Interviews were analyzed thematically. Transcripts were coded manually, and from these codes, themes were built up, both within the groups of interviewees (patients, nurses, TB managers, and shop personnel) and across these groups. Both similarities and differences between interviewees’ responses were noted and divergentviews were described (these were views that did not fit with the general trend of the findings, but that needed to be considered and accounted for in the final explanation) [19]. Transcripts of interviews were read and re-read so that recurring ideas (meaning units) could be identified. Abstractions (codes) of these meaning units were developed, and categories of codes built up into subthemes and themes [19]. For each key analytic theme, data extracts were identified on the basis of being representative and/or interesting illustrations of an emerging issue. The process of data analysis is illustrated in Table 2. Analysis was undertaken by the principal investigator and one of the co-investigators, with contributions from the research assistants who were involved in data collection. Analysis was informed by the principal investigator’s knowledge of the setting and of TB care. However, no formal theoretical stance was taken.

**Table 2:** Example of the analytic process

**Meaning unit**	**Code**	**Sub-theme**	**Theme**
‘It helps me as I say that when I receive the voucher I am able to buy food and take my tablets on the right time; I have to eat before I swallow my tablets that is how the voucher is helping me.’	Voucher helps with buying food with which to take tablets	Important role of voucher is to prevent having to take tablets on empty stomach	Role of voucher in improving adherence
‘… you know that you can only get the voucher while you are on treatment. The minute you are out, it’s back to square one, and those needs that were catered for by those vouchers are now left hanging and the patient might be tempted to default treatment so that to continue getting these vouchers.’	Patients may want to remain ill in order to continue receiving voucher	The voucher may act as a perverse incentive	Negative effects of voucher

### Ethical considerations

Ethical approval for the trial and its process evaluation was received from the Committee for Human Research at the University of Stellenbosch (reference N07/10/245). The trial was registered with Current Controlled Trials (reference ISRCTN50689131), the South African Clinical Trials Registry (reference DOH-27-0409-2791), the Wellcome Trust Register of Clinical Trials (reference 083619), and the Pan-African Clinical Trials Registry (reference PACTR2010010001275437).

Written informed consent was obtained from all participants in the language of their choice (either isiZulu or English). Patients were assured that non-participation in the interviews would not impact on the treatment they received from the clinic, nor on their continued participation in the trial. All participants were assured of confidentiality and no participants were paid for taking part in these interviews.

The hard copies of interviews and participants’ consent forms were stored by the principal investigator in locked drawers. Electronic copies of interviews were stored on the password-protected computer of the principal investigator.

## Results

The intention-to-treat results of the trial showed that although patients who received vouchers had a moderately higher treatment success rate than those in control clinics, this was not statistically significant. Part of the reason for this was the low fidelity to the intervention protocol: of the 2076 patients who were eligible to receive a voucher for the six to eight months of their treatment, 813 (36.2%) did not receive a voucher at all, and 671 (32.3%) received a voucher for between one and three months. The remainder received a voucher for four to eight months of treatment.

This process evaluation shows that the low fidelity occurred for reasons of both principle (participants’ perceptions of equity and social justice) and process (the logistics of voucher administration).

### Equity and social justice in the implementation of the voucher system

The perceptions of all participants were that it was operationally feasible to administer the voucher to patients as a routine part of the TB Control Programme. However, many patients and nurses believed that vouchers should not be given to patients who were relatively better off financially. Nurses noted that it seemed unfair not to give vouchers to patients who were more deprived even if they did not meet the clinical eligibility criteria, and equally unfair to give to those who, for example, were employed or in receipt of a social grant but did met the eligibility criteria. One nurse commented that:

*‘I would decide by looking at who is needy, considering their social problems, then I will give … like if they do tell me they can’t take pills on an empty stomach then I will give considering their background…but those who are working I wouldn’t give …’* (Nurse, Clinic 7 page 3).

Nurses felt that it was not difficult to assess the patients’ levels of need:

*‘It’s easy (to decide who is needy)… By history taking I can ask them, how many members of the family, who is a breadwinner, who is working, then I can assess from there’* (Nurse, Clinic 8 page 4)*.*

The patients themselves felt that this was fair - if they were doing well financially that month, it was better that someone else received the voucher that they might have taken:

*‘Sometimes I do not ask for it, if I see that I do have something’* (Patient, Clinic 5 page 2).

Furthermore, patients, nurses, and shop personnel were concerned that only certain patients (those with pulmonary TB) were eligible to receive the voucher. They felt that this was unfair, given the depth of deprivation experienced by some patients:

*‘I do hear them saying that the voucher is given to those with PTB [pulmonary TB] only … They are complaining - you know people they also want to get the voucher, they are not happy that some people are receiving it while others don’t’* (Mother of 5-year-old patient, Clinic 7 page 4).

Even one cashier was distressed by this exclusivity:

*‘If you could also try and help the others not just the TB patients. We also have people sick besides (those) diagnosed with TB’* (Cashier, Shop 4 page 5).

Managers of the TB program also perceived this as a problem, not for the reasons of equity cited by patients and nurses, but because they feared that it would act as a perverse incentive:

*‘If you target for example the TB patients, then people see that TB patients have better or get things for just being TB patients. So now everyone will want to have TB to get those things especially if they are in need, like food and all those things’* (TB Manager page 4).

*‘… you know that you can only get the voucher while you are on treatment. The minute you are out, it’s back to square one, and those needs that were catered for by those vouchers are now left hanging and the patient might be tempted to default treatment so that to continue getting these vouchers’* (TB manager, page 2).

It was clear from the interviews that all participants found the exclusivity of giving vouchers only to a select group of patients to be problematic. The targeting of economic support to certain groups to the exclusion of others is an important issue in addressing the social determinants of health. How participants are selected for any program that seeks to address poverty-related risk factors for disease may be contentious among participants and administrators of the program. The perceptions of those who implement economic interventions to improve health outcomes, and of those who receive these interventions, may have profound effects on the process of implementation and the effectiveness of such interventions. In this trial, such perception lead nurses to limit the number of vouchers received by eligible patients because they felt that some patients did not need vouchers at all and some needed them only intermittently. The effect that this had on the fidelity to the intervention protocol may have undermined the impact of the voucher and contributed to the non-significant findings of the trial.

### Logistical issues in the implementation of the voucher system

Logistical reasons for patients not receiving vouchers every month included that the vouchers were sometimes not available (because they had not been delivered on time to the clinic by investigators) and that some nurses only distributed vouchers at the end of the month.

Although the investigators visited the participating clinics every four to six weeks, clinics would sometimes run out of vouchers before these visits. Sometimes nurses would only notify the investigators of this problem on the day that the vouchers ran out, and it was not always possible to deliver voucher books to clinics quickly enough to prevent some patients from leaving the clinic without vouchers. Patients commented that:

*‘Sometimes it happens that you come for the voucher and find there are no vouchers. They say come on the following or the third day’* (Patient, Clinic 7 page 3).

In addition, some nurses found it logistically easier to issue all vouchers in one batch at the end of the month. Patients were therefore required to return to the clinic at the end of the month to get their vouchers.

Travelling to the clinic on another day to collect their vouchers would have imposed additional costs on patients, and very poor patients may not have been able to afford these additional visits. Similarly, patients who were working may not have been able to take time off work for an additional clinic visit, and patients who were very ill may not have been able to visit the clinic a second time in a month. Although the proportion of patients affected by these factors was not quantified in the trial, this qualitative process evaluation suggests that at least some patients were affected by them.

### Perceived effects of the voucher on adherence to treatment

Nurses felt that the voucher had improved adherence to treatment:

*‘The adherence was excellent! Even if you check their files they were coming monthly now, there was no need to keep on phoning them to come forward for more treatment, they come monthly because they knew that they will get the voucher …’* (Nurse, Clinic 9 page 1).

*‘I saw that it was bringing back the patients, because patients were informing others about the voucher and the defaulters were coming back to the clinic…Adherence has increased even on the strepto [streptomycin] patients, knowing that they’ll get the voucher every month…. Since the voucher we haven’t had a case where someone [was] stopping treatment and then coming back. We haven’t had patients defaulting’* (Nurse, Clinic 8 pages 1 and 2).

Patients also noted that the voucher enabled better adherence to treatment, through addressing barriers to adherence that were related to poverty:

*‘Yes, having no money can have an effect [on adherence]. Since in the clinic we are given a specific date to collect our treatment, if one does not have money on that day that will be the problem… [Also] If you have TB you must take nutritious food and [if] you don’t have money you can’t take that kind of food’* (Patient, Clinic 3 page 1).

The voucher was seen by patients to have alleviated some of these problems:

*‘Yes it has made it better for me to take my pills, we were so happy about receiving it, food is a problem’* (Patient, Clinic 7 page 5).

‘*It truly made it easier. I get motivated to take my treatment and I can’t wait for my date to go to the clinic for my treatment.*’ (Patient, Clinic 8 page 1).

### Impacts of the voucher on patient poverty

The effects of the voucher on household food expenditure and, by implication, on household food security, will be reported elsewhere. However, in this qualitative process evaluation many patients, nurses, and shop personnel noted that the value of the voucher was small:

*‘It does help me for the time being. I think food for R120 is too small, but I appreciate what they are giving me … the vouchers are small, the voucher is for us to eat for few days, and it’s finished’* (Patient, Clinic 5 pages 1 and 5).

From the perspective of patients, the main value of the voucher lay in providing food with which to take their TB medication. A major theme that emerged from patient interviews was that it was very difficult to take TB tablets on an empty stomach. Doing so made many patients feel very hungry and it made others feel ill. For others, eating before taking their tablets was seen to increase the efficacy of the treatment:

*‘Just imagine as I have said how difficult it is to take them on an empty stomach. In fact it seems as if they are not helping you here but they are killing you!’* (Patient, Clinic 7 page 6).

*‘If you take the tablet without eating anything that tablet will not work - where is it going to stay? It’s better if you got something to eat first’* (Mother of 5-year-old patient, Clinic 7 page 4).

In many cases, patients shared the food purchased with their vouchers with their families as it was considered to be against the norms of family behaviour to eat it alone:

*‘Yes I have to share with them I cannot be able to eat alone … We are a family I cannot be able to eat by myself, I wasn’t taught like not to share’* (Patient, Clinic 7 page 4).

However in some cases, especially in the case of children or the elderly, the food was reserved for the index patient alone.

*‘It’s for the child … This money is hers, the voucher belongs to her as she is taking the treatment’* (Mother of 5-year-old patient, Clinic 7 page 5).

The view that the voucher enabled patients to buy more food with which to take their tablets was echoed by many participants. However, the practice of sharing this food may have impacted on the benefit that the index patient derived. If the economic support intended for one patient only is shared with family or community members, the efficacy of that support may be diluted. The targeting and sharing of economic support for the improvement of health outcomes is an important area for further research.

### Economic assistance to improve health outcomes

Given the study context of widespread poverty, nurses did not see the principle of ‘paying patients’ to behave in a healthy way as problematic:

*‘It’s like we are supporting them [the patients] and it’s a good thing because people don’t have jobs so I don’t see it as paying them, rather as supporting needy people’* (Nurse, Clinic 8 page 3).

However, managers of the TB program had a rather more complex approach to the principle of economic assistance to improve health outcomes. On the one hand, they were supportive of the idea of financial support for TB patients and also felt that it was important to address poverty in patients with TB:

*‘If we just leave those patients [don’t materially support them] chances are we will get them coming back again with the disease… I think if we address poverty, if we improve the living conditions of people, then we will go a long way towards addressing TB’* (Senior TB Manager page 2).

However, they were concerned about creating dependency on the vouchers and providing a perverse incentive to remain ill:

*‘We have had cases where patients will sell their sputum… Ja, we’ve had cases where they will sell their TB counts and cards and move into other clinics so that they can be considered to be on treatment and then, so that they can get [the disability] grant. So it does create dependence and there are loops within the system that make it vulnerable to misuse…’* (Senior TB Manager page 3).

Although it did not seem to detract from her support of the continuation of the voucher, one nurse echoed this fear:

‘*The way people are depending on the money, some want to be sick’* (Nurse, Clinic 8 page 3).

A related concern of one senior TB manager was that receipt of the voucher would make it unnecessary for patients to work:

*‘The reason why people go for those grants is to get something to support their families….. So I see it as perpetuating the culture of not working because they’re relying on the grant maybe… we don’t want to create a situation where people are dependent on some monetary incentive somewhere’* (Senior TB Manager page 2).

These complex views reflect both the perceptions of the deep poverty which prevails in KwaZulu-Natal and the effects of poverty on illness, as well as ideas on how poverty should be addressed. These views echo age-old debates over social support for the poor and are discussed further below.

### Leakage and misuse of the vouchers

The terms ‘leakage’ and ‘misuse’ are used variably by studies investigating the use of vouchers to incentivize behaviour change [20]. In this paper, the term ‘leakage’ refers specifically to the receipt of the voucher by groups not eligible to receive it, whilst ‘misuse’ is a broader term which encapsulates the concept of leakage and includes the theft or fraudulent acquisition of a voucher, as well as inappropriate use of the vouchers, for example on cigarettes or alcohol (to be reported elsewhere).

The ‘leakage’ of vouchers to those for whom they were not intended was closely monitored in this study. The investigators were able to correlate all vouchers issued by the clinics with those redeemed from the shops.

Generally, patients, nurses, and shop personnel were happy that, on some occasions, relatives or friends of voucher recipients redeemed the vouchers on their behalf. However, there were three reports from interviewees of cases where patients were not given their vouchers, or the goods redeemed with their vouchers, by the people who had collected them:

*‘… we have the Nompilo’s [lay DOT supporters] … there is a lot of cruelty going on, they act like they are coming to get it [the voucher] for you when they take it themselves and you will feel bad, so it’s better to come and get it yourself’* (Patient, Clinic 7 page 6).

*‘only exceptional … case where I discovered that one patient complained that some of the family members were taking the food … but only one patient … we could rectify that mistake’* (Nurse, Clinic 9 page 1).

One shop owner said that a stolen voucher had been brought in to be redeemed:

*‘One case happened when a voucher was stolen but we managed to sort that one out ….I can’t remember clearly what happened but the story from the cashiers was that someone came even before the voucher was redeemed and said his voucher has been stolen and told them his name. When the person who stole the voucher came through he was asked his name and proof of that name and he left the voucher there and never came back’* (Owner, Shop 5, page 2).

There were no occasions where the theft or loss of a voucher had to be investigated, or where the issuing of vouchers had to be stopped due to such problems.

## Discussion

This qualitative process evaluation provides contextual and process information which goes beyond the trial design and methodology to explain the trial findings [15]. Importantly, it sheds light on some of the reasons for the poor fidelity to the intervention protocol of the trial, and in so doing, explains to some extent why the effects of the intervention were small. It also provides insights into the perceived value of economic support in improving TB treatment outcomes, as well as into participants’ broader views on the concept of economic assistance for the improvement of health outcomes. These findings may help to inform further research as well as the large scale implementation of similar interventions, in other contexts [15]. There were two important factors that detracted from fidelity to the intervention protocol: nurses’ perceptions of the inequity of the criteria for receiving vouchers; and logistical issues involved in voucher administration. Nurses modified the delivery of these vouchers in several ways but, perhaps most importantly for this trial, they rejected the eligibility criteria as being unjust. Working in a context of widespread and deep poverty, it seemed unfair to them to give vouchers only to those who met the eligibility criteria when some of those who met the criteria needed the voucher less and some who didn’t meet the criteria needed it more. Although the eligibility criteria for receipt of a voucher (which did not include socioeconomic status) were clearly outlined to nurses at the start of the trial, the imperative to ration the vouchers was stronger. Nurses frequently ration the food parcels that may be provided to needy patients as part of their routine TB treatment because there is usually insufficient food to give to every patient, and they seemed to treat the vouchers in the same way. This rationing impacted on the delivery of the vouchers and reaffirms the importance of the ‘street level bureaucrat’ as an implementer [21]. Lipsky [22] suggests that ‘street level bureaucrats’ (public service workers who have important roles in delivering government services, have constant interaction with members of the public, and are able to use their own discretion in carrying out their activities) are not passive media through which the policy or intervention passes from the designers to the recipients. On the contrary, implementers can be seen as active in interpreting and, if considered necessary, modifying policies or interventions, to the extent that they can may constitute a level of policy-making themselves [22]. Logistical problems also impacted on fidelity to the delivery of the intervention. In some clinics it was administratively easier for nurses to give out all the vouchers at the end of the month, but this required patients to come back to the clinic at this time to receive them. Because the study team was small, there were also logistical difficulties with delivering vouchers to all clinics on time. Voucher books were personally delivered to all clinics and collected from all shops by the principal investigator and one assistant, with staff at clinics and shops required to sign proof of delivery or collection. This meant that vouchers were sometimes not available for patients on their appointment dates, which necessitated another visit to the clinic to collect them. Although the frequency of these occurrences were not quantified and seemed a less important barrier to the implementation of the vouchers than the nurses’ rationing, they were raised by some interviewees in this process evaluation. Such logistical issues should be considered in the replication of similar interventions in other settings. The coordination of delivery of vouchers to clinics and collection of vouchers from shops requires considerable organization and a dedicated staff complement. Setting up the infrastructure to manage the voucher system may be difficult where health systems are weak and resources very limited.

Related to issues of logistics are mechanisms for control of the distribution and use of the voucher. There were very few cases of leakage of the vouchers to those for whom they were not intended. In only one case was the theft of a voucher reported and even in this case the theft was reported before the voucher could be redeemed. More common was the fear that lay Directly Observed Treatment Supporters (DOT supporters) [23] who might collect vouchers on behalf of patients who were too ill to collect them themselves, would either not give the vouchers to the patients or would buy food for themselves. Even this, however, was expressed by only a few participants. The level of leakage of vouchers in this study was thus very small compared to the levels experienced at provincial and national levels with other social grants. For example, in a different province of South Africa, 3000 cases of social grant fraud were recently handed over to the Special Investigating Unit for prosecution. Although it is possible that cases of leakage would increase if vouchers were delivered on a larger scale, the system used in this study is promising in terms of minimizing that potential.

Like patients in Mexico’s conditional cash transfer program [24], we found that many patients shared their voucher purchases with their families, and that this was consistent with their social values. Although this may have diluted the effect of the vouchers for the index patients, for many it was inconceivable that they should keep the voucher to themselves. Like other social grants in South Africa, the material benefits of the grant are generally distributed throughout the household, so that the household is the unit that benefits rather than the individual recipient [25]. Therefore, for this voucher (as for other social grants) it is important to note that the impact of policies targeted at individuals will be mediated by household dynamics [26]. If the value of the voucher had been larger the dispersion of this benefit may have improved the nutritional status of other household members and so reduced their risk of contracting or developing TB [27-29]. However, because it was relatively small these sharing practices may have meant that neither the index patients nor their households could benefit maximally from the vouchers.

Nurses were of the view that patients who received the voucher came back to the clinics to collect their tablets regularly, and that even known defaulters returned to the clinic to resume treatment. One of the reasons for the perceived improvement in adherence is that most patients who were interviewed valued the food purchases that they could make with their vouchers, and that this food helped to avoid having to take tablets on an empty stomach. This is consistent with the ‘enabling’ effect of economic support to poor patients who are ill; that is, that such support enables adherence by minimizing some of the barriers to adherence imposed by poverty.

This qualitative process evaluation showed that patients generally felt that the value of the voucher (R120.00) was small, and indeed it was, compared to the Child Support Grant (valued at R250.00 per month at the time of the trial), the Old Age Pension, and the Disability Grant (both valued at R1080.00 per month at the time of the trial). However, it was about a fifth of the value of the median per capita income in KwaZulu-Natal around the time of the trial [30], and most participants said it was helpful, particularly in enabling them to buy the food to take their tablets with. This was a powerful theme in the patients’ interviews, with most saying that it was impossible to take tablets on an empty stomach. In this sense, the voucher enabled patients to take tablets where this may have been difficult without it.

Acknowledging the link between poverty and TB, the TB managers and nurses interviewed in this study agreed in theory with the principle of social assistance for people who are poor and ill. However, managers raised concerns about the impact of a financial transfer to patients with TB in terms of the development of dependency on the grant which is a widespread concern in the country [31]. However, this fear was not realized in our study, which suggested that the more often patients received vouchers the more likely they were to achieve treatment success [12]. It is interesting to note, however, that concerns around dependency on grants and their perverse incentive effects go back a long way in South Africa’s history, and were important debates at the time of the Lund Commission in 1995 [16] and the Carnegie Commission in 1932 [17]. These concerns persist today and relate largely to the concept of the ‘deserving poor’, a concept first articulated in the Poor Laws of Elizabethan England and they remain an area of debate for welfare states today [32]. In essence, the poor who deserve assistance from the state are felt to be those who are unable to work, such as the very young, the elderly, and the disabled. However, in current day South Africa the opportunities for formal employment are diminishing and those which are available are increasingly for people with a completed secondary or tertiary education [33]. Social grants are an important, perhaps even a crucial, means of survival for the poor in this country [34]. Further research is therefore needed to explore whether these perverse effects are indeed found in practice in order to inform ongoing debates.

## Conclusions

This qualitative process evaluation of a cluster randomized controlled trial provides evidence of the importance of contextual factors in influencing the implementation and effectiveness of economic support to patients with TB in South Africa. The delivery of a voucher to enable better adherence to TB treatment was well received by patients, health workers, and shop personnel in this trial. Both patients and nurses felt that it improved adherence, particularly patients, who felt that the voucher enabled them to buy food with which to take their tablets, thereby making treatment taking easier.

However, a number of factors limited the reach of the voucher to all eligible patients. Some of these factors were a consequence of the beliefs and values of the nurses who distributed them and others were logistical. Nurses felt strongly that vouchers should be given out on the basis of need rather than on trial eligibility, and this is likely to have reduced the extent to which the intervention was delivered as intended. Administrative difficulties that may have impacted on fidelity included a failure on the part of investigators to ensure that all clinics had an uninterrupted supply of vouchers, and a preference in some clinics for giving out vouchers in batches at the end of the month instead of at the time of the patients’ appointments. These factors explain some of the reasons for the non-significant results of this trial and must be addressed in further research around such interventions.

Finally, managers of the TB Control Programme raised concerns about the development of dependence on the vouchers and a perverse incentive effect. Although neither of these fears were realized in our trial, and are not supported by data from other studies, they are deeply felt by many in South African society and have been prevalent in the country since early on in the development of its social welfare system.

Although enough is known about the association between poverty and TB to justify action [1], there is very little evidence on what type of action works best in different contexts. Available evidence from randomized controlled trials is centered in the United States [11] and may not be applicable to middle- and low-income countries where the burden of TB is highest. More research on existing economic interventions, as well as further trials on new types of such interventions, are important to ascertain how best to deliver economic support to those suffering from, and susceptible to TB.

## Abbreviations

DOT supporter: Directly Observed Treatment Supporter; DOT(S): Directly Observed Treatment Short course. A strategy promoted by the World Health Organization for TB control; HIV: Human Immunodeficiency virus; TB: Tuberculosis.

## Competing interests

The authors declare that they have no competing interests.

## Authors’ contributions

EL conceptualized the process evaluation, designed the data collection tools, monitored data collection, wrote the analysis plan, cleaned the data, analyzed the data, and drafted and revised the paper. SL conceptualized the process evaluation, designed the data collection tools, wrote the analysis plan, analyzed the data, and drafted and revised the paper. JV conceptualized the process evaluation, designed the data collection tools, wrote the analysis plan, and drafted and revised the paper. All authors read and approved the final manuscript.

## Supplementary Material

Additional file 1 Consort checklist for trial. Click here for file
